# Supplementary material for: Epidemiological and clinical characteristics of Peruvian patients with mpox: A systematic review and meta-analysis
Source: PLoS One. 2025 Jun 25;20(6):e0327097. doi: 10.1371/journal.pone.0327097 (PMC12194101; doi:10.1371/journal.pone.0327097)
Supplement: S2 Table — (DOCX) [file pone.0327097.s002.docx]

**Table S2.** The adjusted search terms as per searched electronic databases or search tools.

| PubMed | | | |
| --- | --- | --- | --- |
|  | #1 | Monkeypox[Title/Abstract] OR "Mpox"[Title/Abstract] | 4,972 |
|  | #2 | Peru[Title] OR Peruvian[Title] OR Lima[Title] | 10,200 |
|  | #3 | #1 AND #2 | 12 |
| Scopus | | | |
|  | #1 | TITLE( Monkeypox OR Mpox) | 4,687 |
|  | #2 | TITLE(Peru OR Peruvian OR Lima) | 36,532 |
|  | #3 | #1 AND #2 | 15 |
| Embase | | | |
|  | #1 | ('monkeypox'/exp OR 'mpox'/exp).ti,ab. | 5,561 |
|  | #2 | (' Peru' OR 'Peruvian' OR 'Lima').ti,ab. | 37,023 |
|  | #3 | #1 AND #2 | 17 |
| Web of Science | | | |
|  | #1 | TI=(Monkeypox OR Mpox) | 4,340 |
|  | #2 | TI=(Peru OR Peruvian OR Lima) | 26,365 |
|  | #3 | #1 AND #2 | 11 |
| ScienceDirect | | | |
|  | #1 | Title, abstract, keywords: ( Monkeypox OR Mpox) | 1,685 |
|  | #2 | Title, abstract, keywords: ( Peru OR Peruvian OR Lima ) | 10,362 |
|  | #3 | #1 AND #2 | 6 |
| Google Scholar | | |  |
|  | #1 | allintitle: "Monkeypox OR Mpox" | 9,540 |
|  | #2 | allintitle: "Peru OR Peruvian OR Lima" | 311,000 |
|  | #3 | #1 AND #2 | 15 |
| Virtual Health Library (VHL) | | | |
|  | #1 | ti:(Monkeypox OR Mpox) | 4,765 |
|  | #2 | ti:( Peru OR Peruvian OR Lima) | 22,626 |
|  | #3 | #1 AND #2 | 15 |
| Scielo | | | |
|  | #1 | (ti:(*monkeypox OR *mpox)) | 87 |
|  | #2 | (ti:(*Peru OR *Peruvian OR *Lima )) | 9 982 |
|  | #3 | #1 AND #2 | 6 |
| Dimensions | | | |
|  | #1 | Title and abstract (monkeypox OR mpox) | 7,694 |
|  | #2 | Title and abstract (Peru OR Peruvian OR Lima) | 157,837 |
|  | #3 | #1 AND #2 | 44 |
| Epistemónikos | | | |
|  | #1 | title:(monkeypox OR mpox) | 1,932 |
|  | #2 | title:(Peru OR Peruvian OR Lima) | 2,583 |
|  | #3 | #1 AND #2 | 9 |
| TOTAL |  | PubMed (n =12); Scopus (n=15); Web of Sciences(n=11); Embase (n=17); ScienceDirect (6); Google Scholar (15); Virtual Health Library (15); Scielo (6), Dimensions (44), and Epistemónikos (9) | 150 |
| Duplicates | | | 103 |
| Records screened | | | 47 |
| Records excluded by title and abstract | | | 22 |
| Reports evaluated by full text | | | 25 |
| Reports excluded by full text | | | 16 |
| Reports included in full text | | | 9 |
