# Supplementary material for: Epidemiological and clinical characteristics of Peruvian patients with mpox: A systematic review and meta-analysis
Source: PLoS One. 2025 Jun 25;20(6):e0327097. doi: 10.1371/journal.pone.0327097 (PMC12194101; doi:10.1371/journal.pone.0327097)
Supplement: S3 Table — (DOCX) [file pone.0327097.s003.docx]

**Table S3**. Quality of the mpox studies included in the review

| Authors | Year | Eligibility criteria | Study subjects and the setting | Exposure measured in a valid and reliable way 'gold standard' | A specified diagnosis or definition | Confounding factors | Dealing with confounding factors | Outcomes measured in a valid and reliable way | Appropriate statistical analysis | Scores (8) | Quality (high, moderate, low) |  |
| --- | --- | --- | --- | --- | --- | --- | --- | --- | --- | --- | --- | --- |
| Ramírez-Soto MC, et al. (1) | | 2024 | Yes | Yes | Yes | Yes | Unclear | NA | Yes | Yes | 6 | Moderate |
| Sihuincha Maldonado M, et al. (2) | | 2023 | Yes | Yes | Yes | Yes | Unclear | NA | Yes | Yes | 6 | Moderate |
| Alfaro Angulo MA, et al. (3) | | 2024 | Yes | Yes | Yes | Yes | Unclear | NA | Yes | Yes | 6 | Moderate |
| Reaño Tovar FM, et al. (4) | | 2024 | Yes | Yes | Yes | Yes | Unclear | NA | Yes | Yes | 6 | Moderate |
| Pampa-Espinoza L, et al. (5) | | 2022 | Yes | Yes | Yes | Yes | Unclear | NA | Yes | Yes | 6 | Moderate |
| Terry Castellano LE, et al. (6) | | 2023 | Yes | Yes | Yes | Yes | Unclear | NA | Yes | Yes | 6 | Moderate |
| Briceño M. (7) | | 2023 | Yes | Yes | Yes | Yes | Unclear | NA | Yes | Yes | 6 | Moderate |
| Araujo-Castillo JF, et al. (8) | | 2023 | Yes | Yes | Yes | Yes | Unclear | NA | Yes | Yes | 6 | Moderate |
| Bonifacio Morales N, et al. [31] | | 2024 | Yes | Yes | Yes | Yes | Unclear | NA | Yes | Yes | 6 | Moderate |

NA: Not assessed

Moola S, Munn Z, Tufanaru C, Aromataris E, Sears K, Sfetcu R, Currie M, Qureshi R, Mattis P, Lisy K, Mu P-F Chapter 7: Systematic reviews of etiology and risk: JBI; 2020. Available from: <https://synthesismanual.jbi.global>
