# Supplementary material for: Epidemiological and clinical characteristics of Peruvian patients with mpox: A systematic review and meta-analysis
Source: PLoS One. 2025 Jun 25;20(6):e0327097. doi: 10.1371/journal.pone.0327097 (PMC12194101; doi:10.1371/journal.pone.0327097)
Supplement: S5 Table — (DOCX) [file pone.0327097.s005.docx]

**Table S5**. Meta-analysis database

| **Variables and Studies** | **Ramírez-Soto MC, et al.** | **Sihuincha Maldonado M, et al.** | **Alfaro Angulo MA, et al.** | **Reaño Tovar FM, et al.** |
| --- | --- | --- | --- | --- |
| Sample | 3561 | 205 | 48 | 124 |
| Male | 3433 | 202 | 47 | 122 |
| Female | 128 | 3 | 1 | 2 |
| Individuals with HIV | 2123 | 136 | 36 | 71 |
| Individuals with HIV on HAART | 1796 | 129 | 36 | 58 |
| Hospitalization | 192 | 21 | 3 | 2 |
| Syphilis | 619 | 67 | 4 | 12 |
| Heterosexual | 821 | 13 | 13 | 40 |
| Homosexual | 2046 | 166 | 28 | 55 |
| Bisexual | 570 | 26 | 7 | 21 |
| Fever | 2317 | 162 | 26 | 82 |
| Headache | 1780 | 119 |  | 63 |
| Myalgia | 1367 |  | 21 | 35 |
| Fatigue or Asthenia | 1095 | 105 | 25 | 25 |
| Local lymphadenopathy | 1307 | 98 | NR | 19 |
| General lymphadenopathy | 209 | 13 | 25 | 9 |
| Lymphadenopathy any type | 3512 | 111 | NR | NR |
| Rash or skin lesions at consultation (Local) | 750 | 38 | NR | 15 |
| Rash or skin lesions at consultation (General) | 2792 | 166 | 48 | 108 |
| Anogenital rash | 2464 | 160 | 33 | NR |
| Proctitis | 2464 | 19 | 3 | 6 |

NR: Not reported
