# Supplementary material for: Epidemiological and clinical characteristics of Peruvian patients with mpox: A systematic review and meta-analysis
Source: PLoS One. 2025 Jun 25;20(6):e0327097. doi: 10.1371/journal.pone.0327097 (PMC12194101; doi:10.1371/journal.pone.0327097)
Supplement: S6 Table — (DOCX) [file pone.0327097.s006.docx]

**Table S6**. R version 4.2.3. script

| **Import database** |
| --- |
| Environment – From Excel – Import Excel Data - Import |
| **Calculate the prevalence of epidemiological and clinical characteristics of mpox** |
| library(meta)  library(readxl)  data_prevalence<- **Database name**  mtprop=metaprop(event= **Epidemiological and clinical characteristics**,  n= **Sample_** **Study**,  studlab=paste(Study,year),  data=data_prevalence,  method.tau = "DL",  method = "Inverse",  method.ci = "SACC",  sm="PFT")  mtprop  forest(mtprop,  comb.fixed=FALSE,  common = FALSE)  **An example is given**:  Database name: **Base_HIV**   \| Study \| year \| **Sample** \| **HIV** \| \| --- \| --- \| --- \| --- \| \| **Ramírez-Soto MC, et al.** \| 2024 \| 3561 \| 2123 \| \| **Sihuincha Maldonado M, et al.** \| 2023 \| 205 \| 136 \| \| **Alfaro Angulo MA, et al.** \| 2024 \| 48 \| 36 \| \| **Reaño Tovar FM, et al.** \| 2024 \| 124 \| 71 \|   **Code in R version 4.2.3**  library(meta)  library(readxl)  data_prevalence<- **Base_HIV**  mtprop=metaprop(event= **HIV**,  n= **Sample**,  studlab=paste(Study,year),  data=data_prevalence,  method.tau = "DL",  method = "Inverse",  method.ci = "SACC",  sm="PFT")  mtprop  forest(mtprop,  comb.fixed=FALSE,  common = FALSE) |
