# Supplementary figures and images for: Epidemiological and clinical characteristics of Peruvian patients with mpox: A systematic review and meta-analysis
Source: PLoS One. 2025 Jun 25;20(6):e0327097. doi: 10.1371/journal.pone.0327097 (PMC12194101; doi:10.1371/journal.pone.0327097)

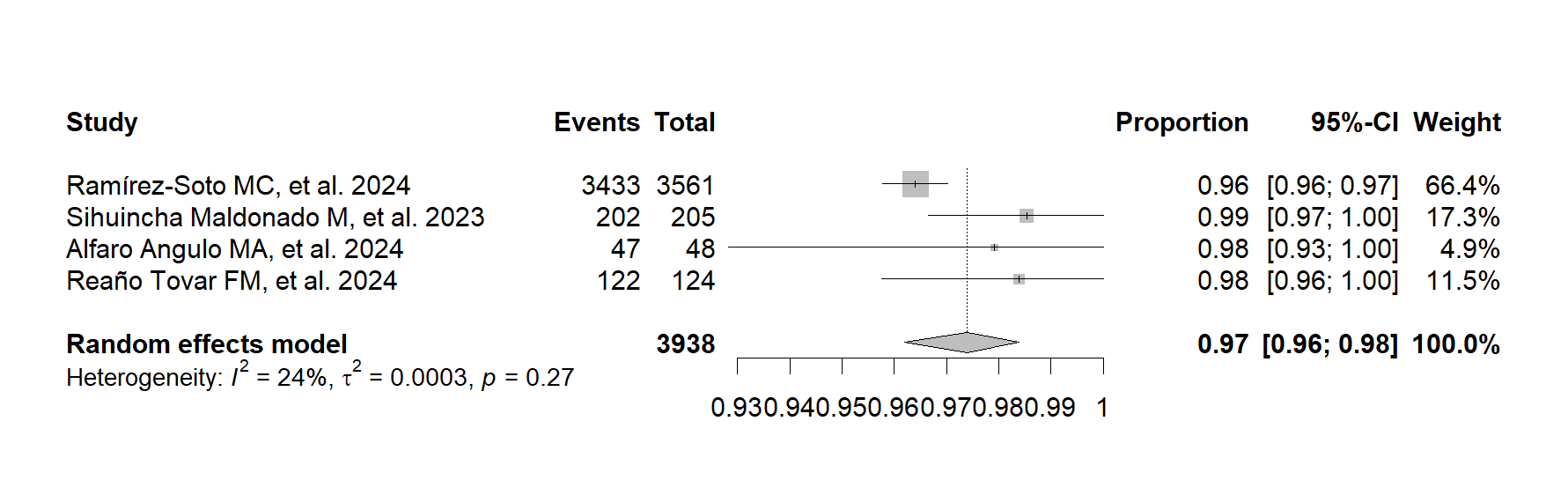

Supplement: S1 Fig — (TIF) [file pone.0327097.s007.tif]

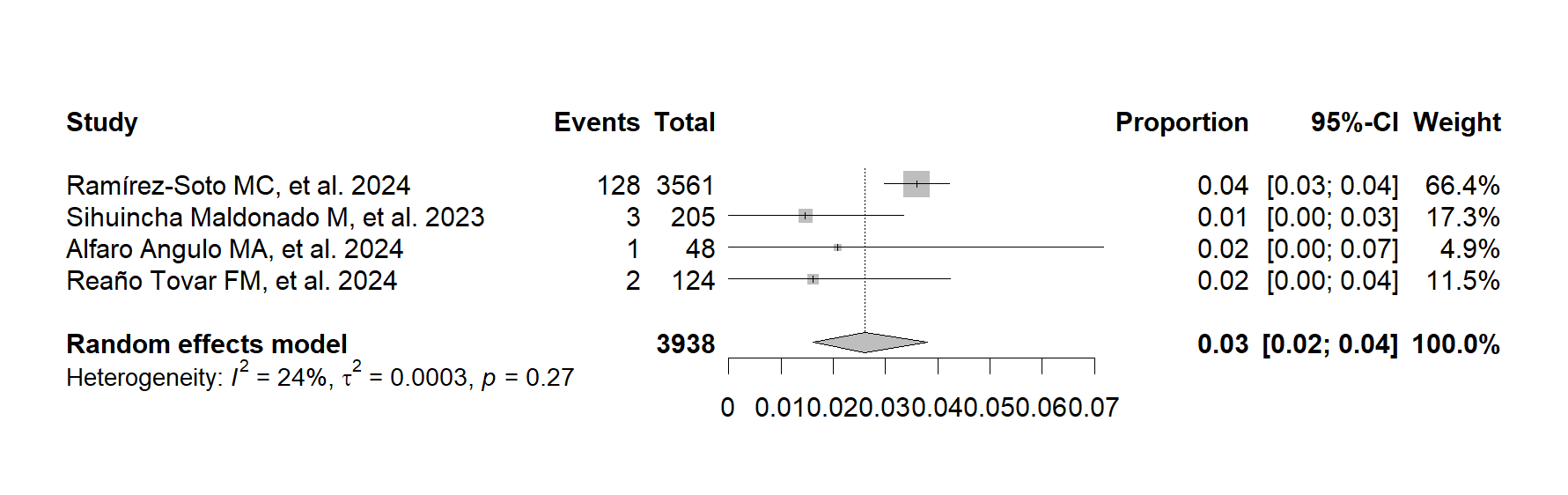

Supplement: S2 Fig — (TIF) [file pone.0327097.s008.tif]

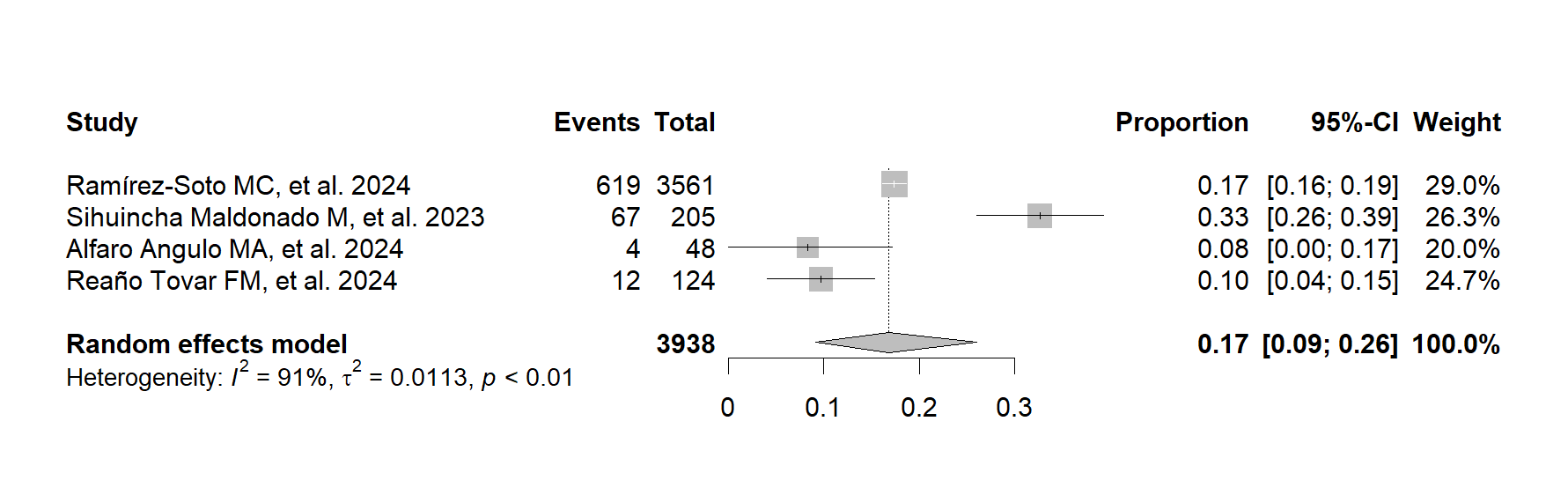

Supplement: S3 Fig — (TIF) [file pone.0327097.s009.tif]

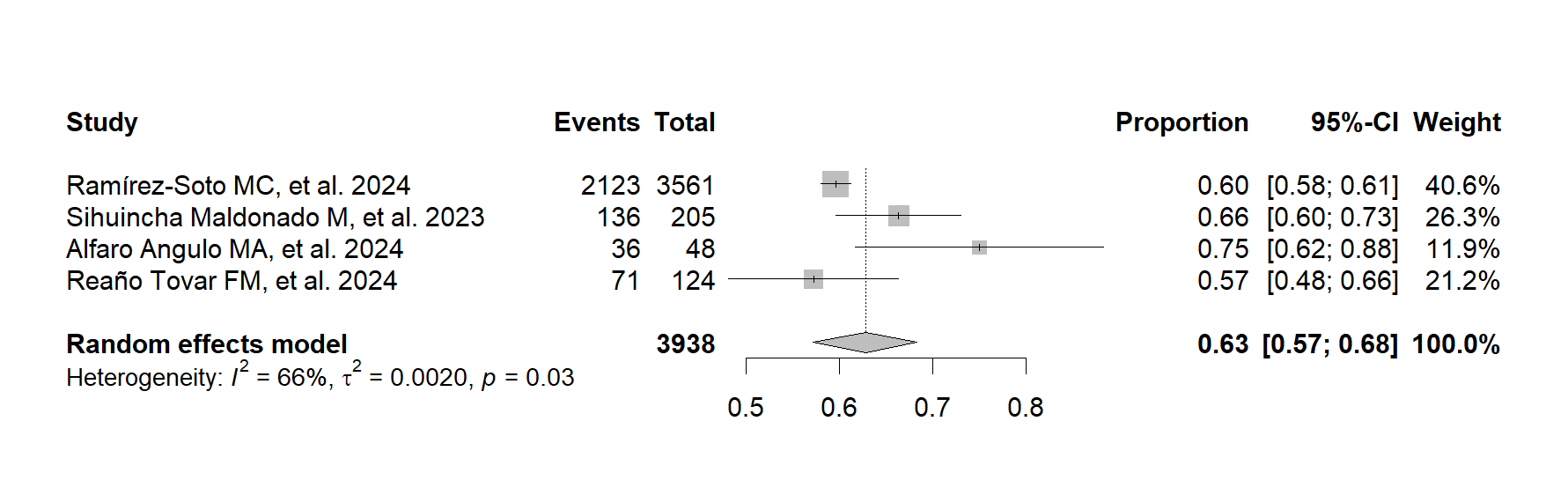

Supplement: S4 Fig — (TIF) [file pone.0327097.s010.tif]

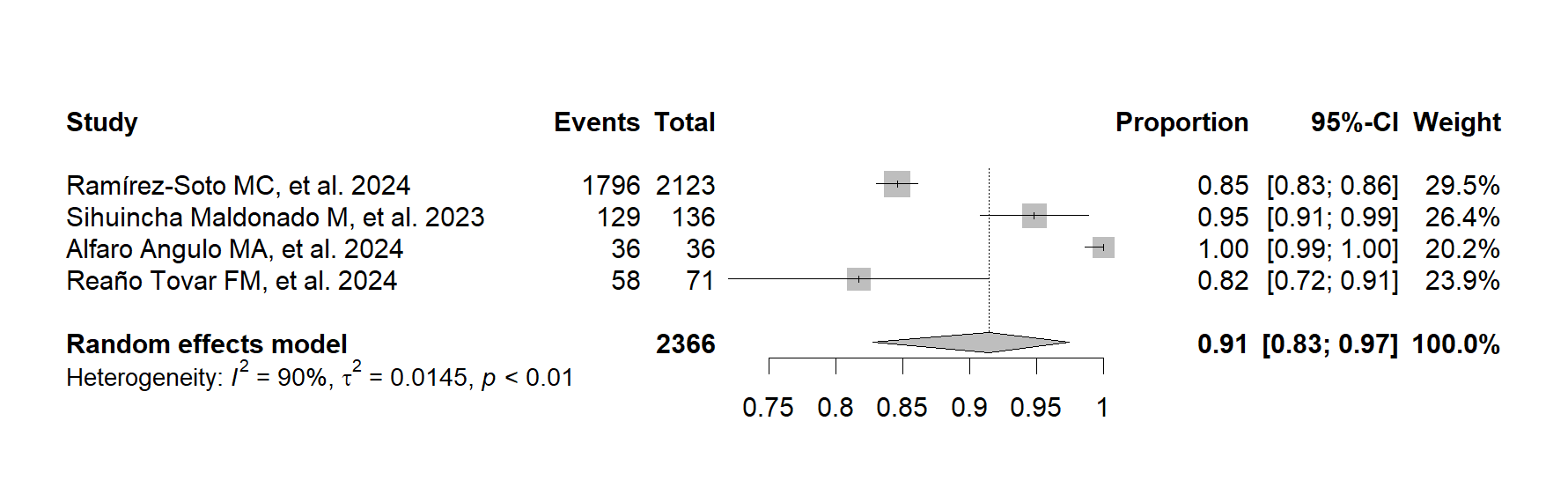

Supplement: S5 Fig — (TIF) [file pone.0327097.s011.tif]

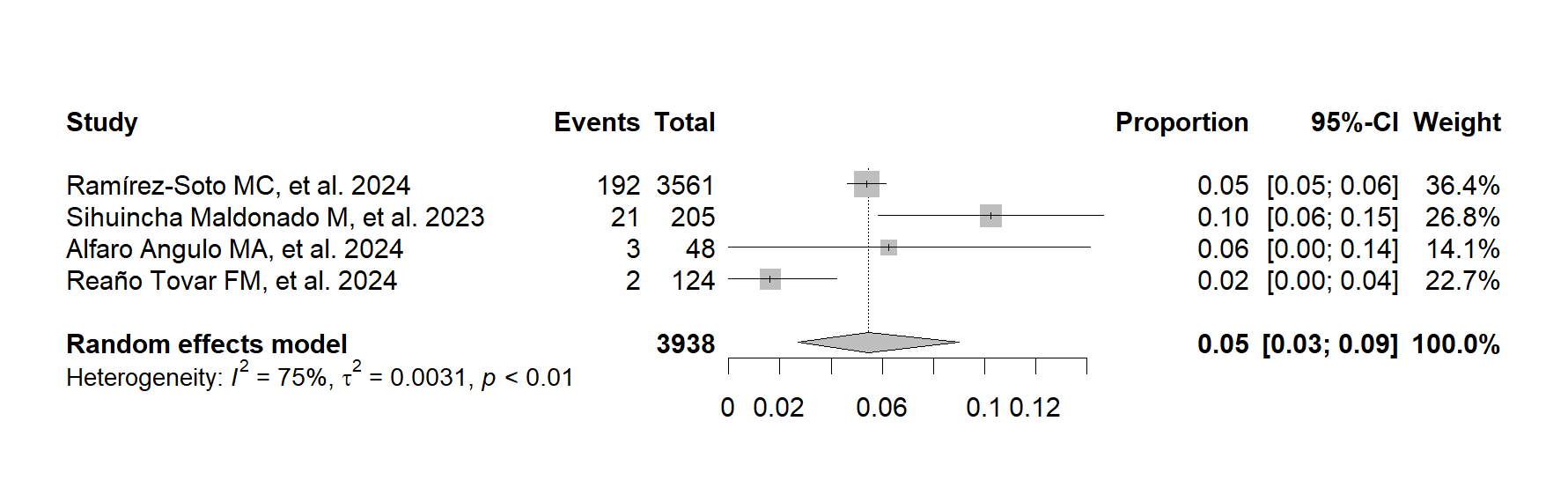

Supplement: S6 Fig — (TIF) [file pone.0327097.s012.tif]

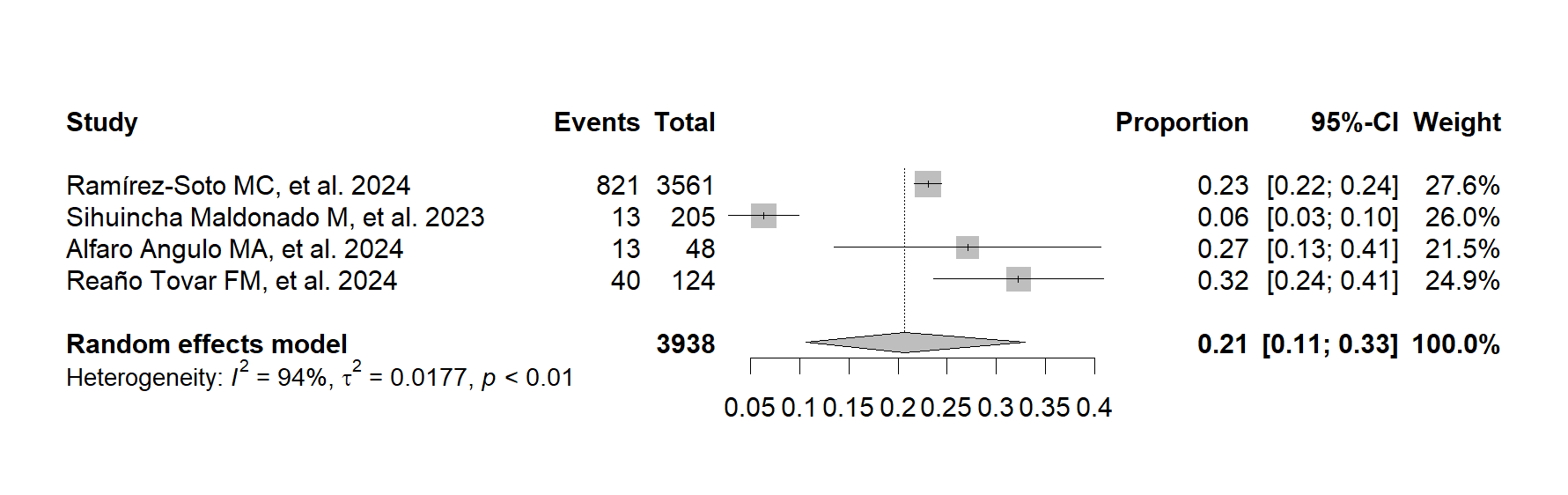

Supplement: S7 Fig — (TIF) [file pone.0327097.s013.tif]

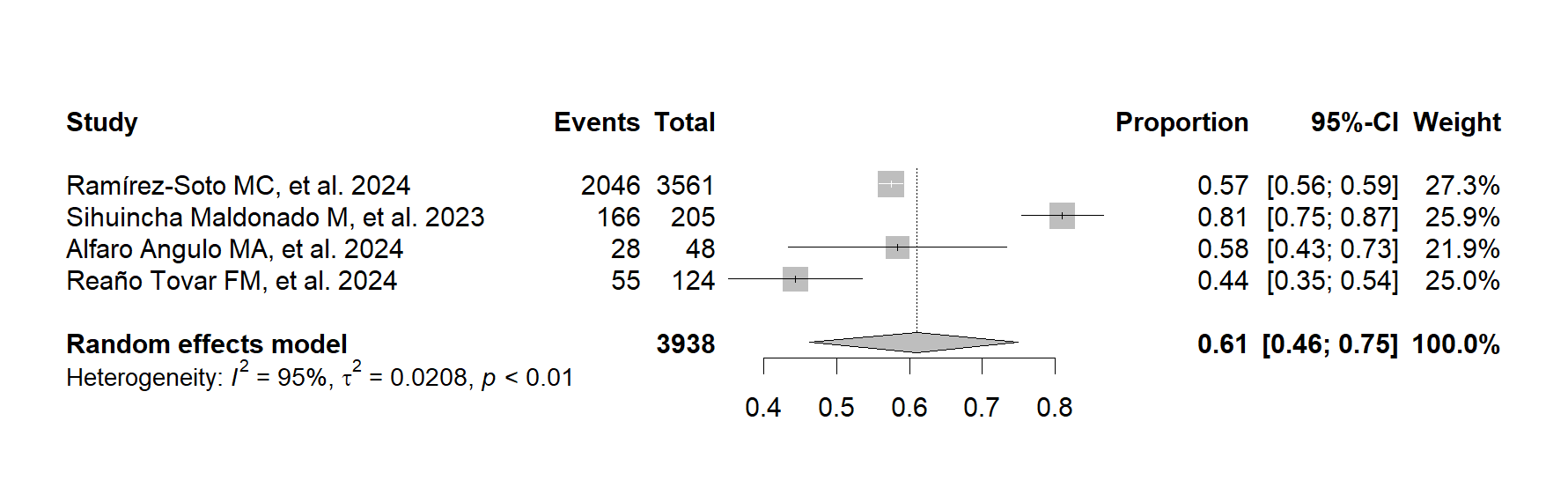

Supplement: S8 Fig — (TIF) [file pone.0327097.s014.tif]

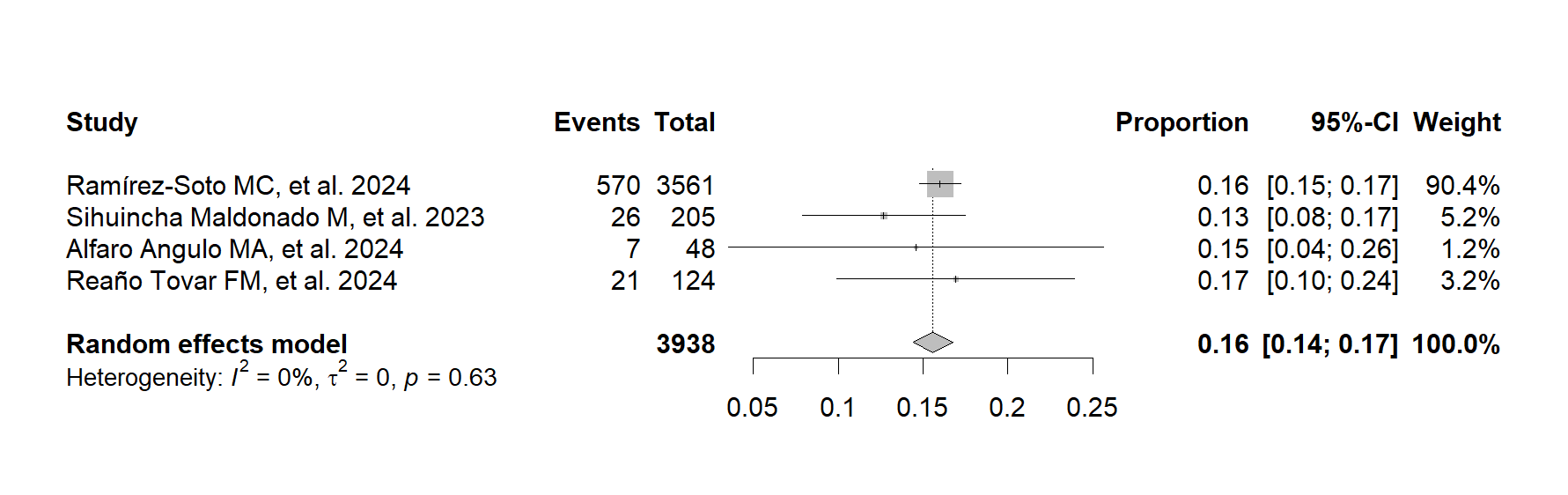

Supplement: S9 Fig — (TIF) [file pone.0327097.s015.tif]

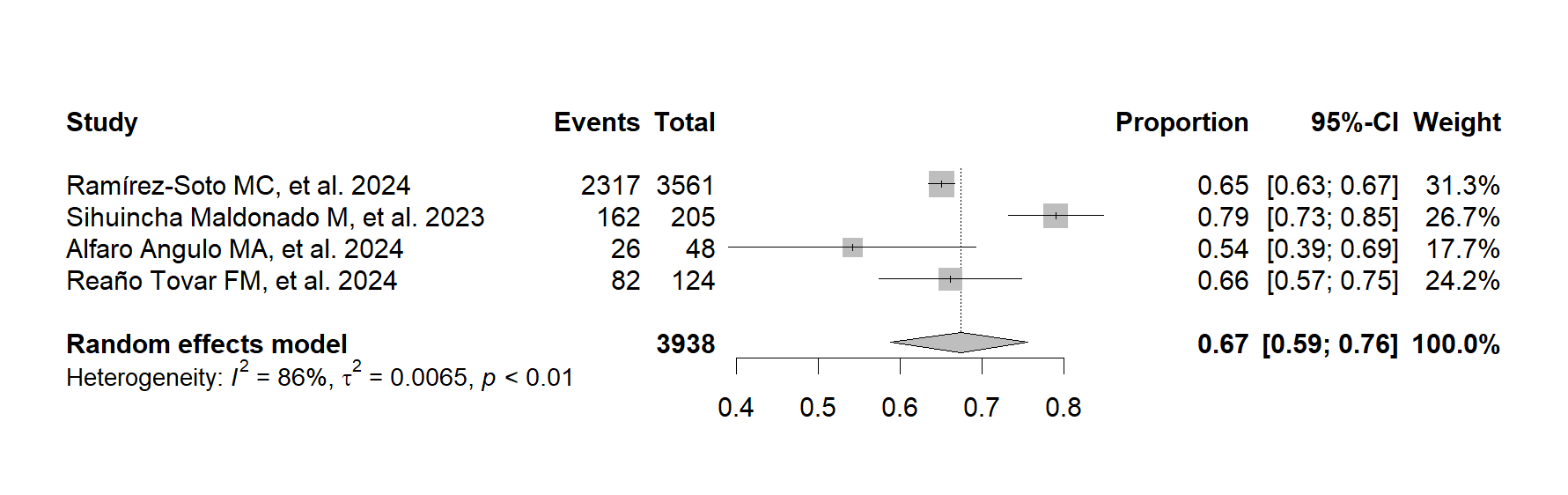

Supplement: S10 Fig — (TIF) [file pone.0327097.s016.tif]

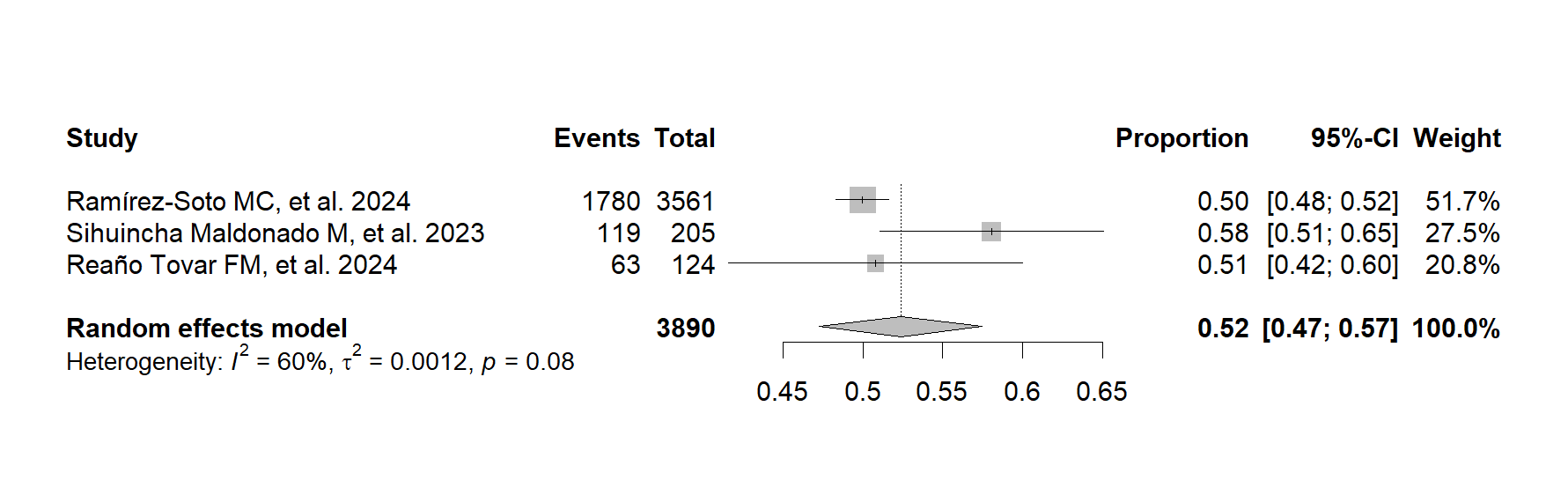

Supplement: S11 Fig — (TIF) [file pone.0327097.s017.tif]

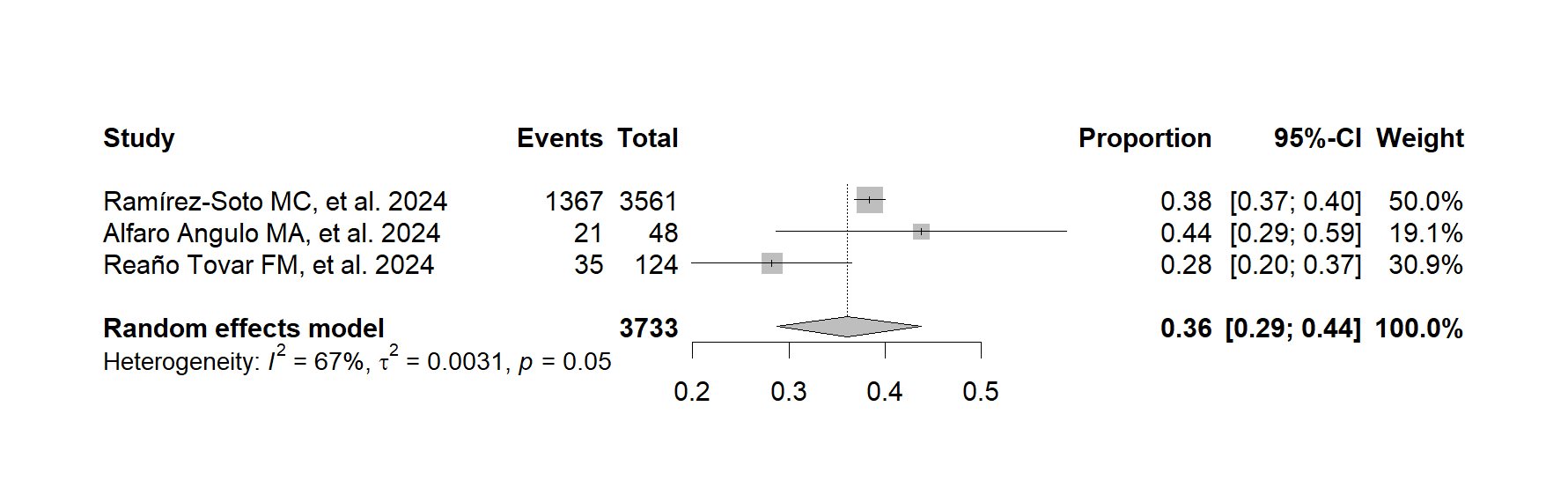

Supplement: S12 Fig — (TIF) [file pone.0327097.s018.tif]

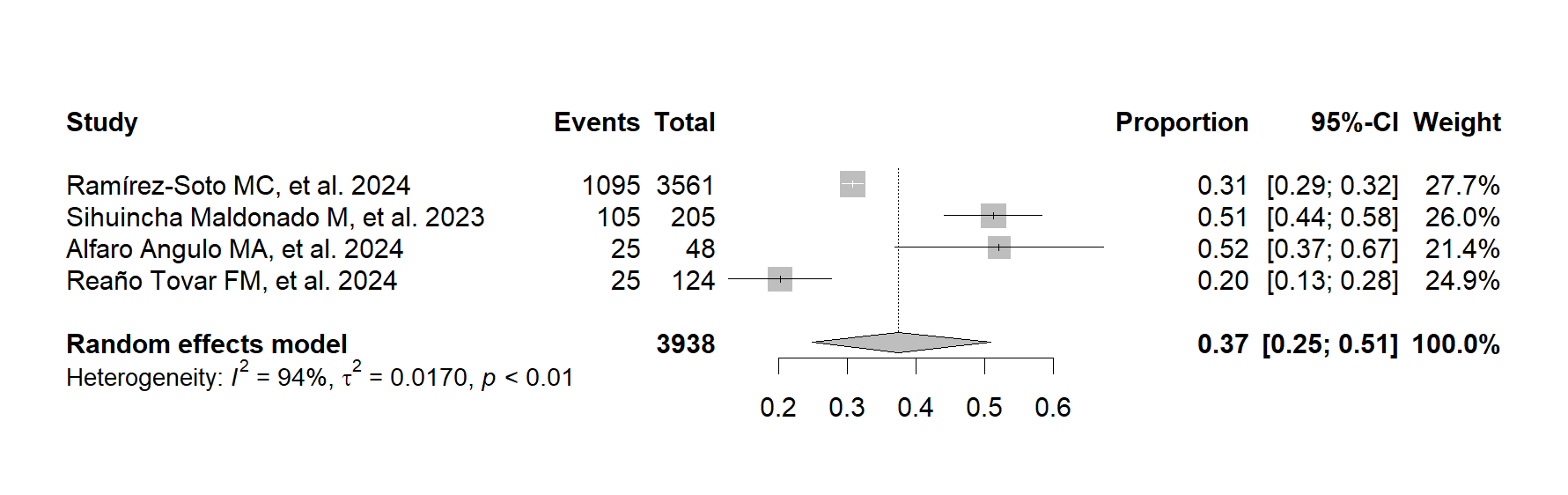

Supplement: S13 Fig — (TIF) [file pone.0327097.s019.tif]

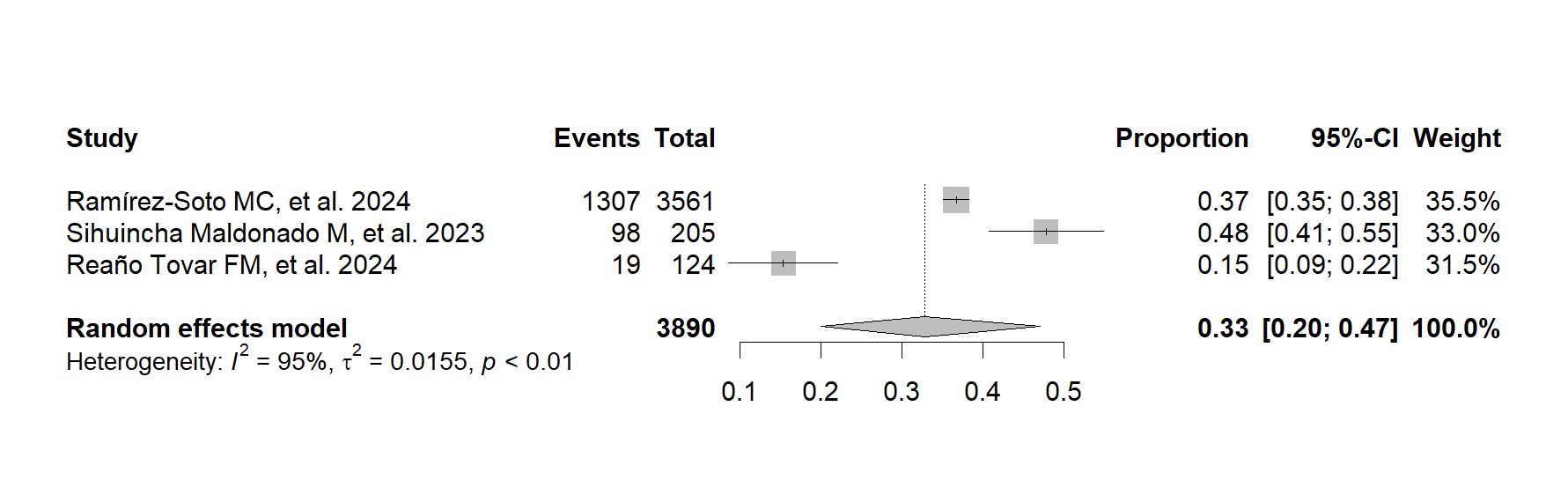

Supplement: S14 Fig — (TIF) [file pone.0327097.s020.tif]

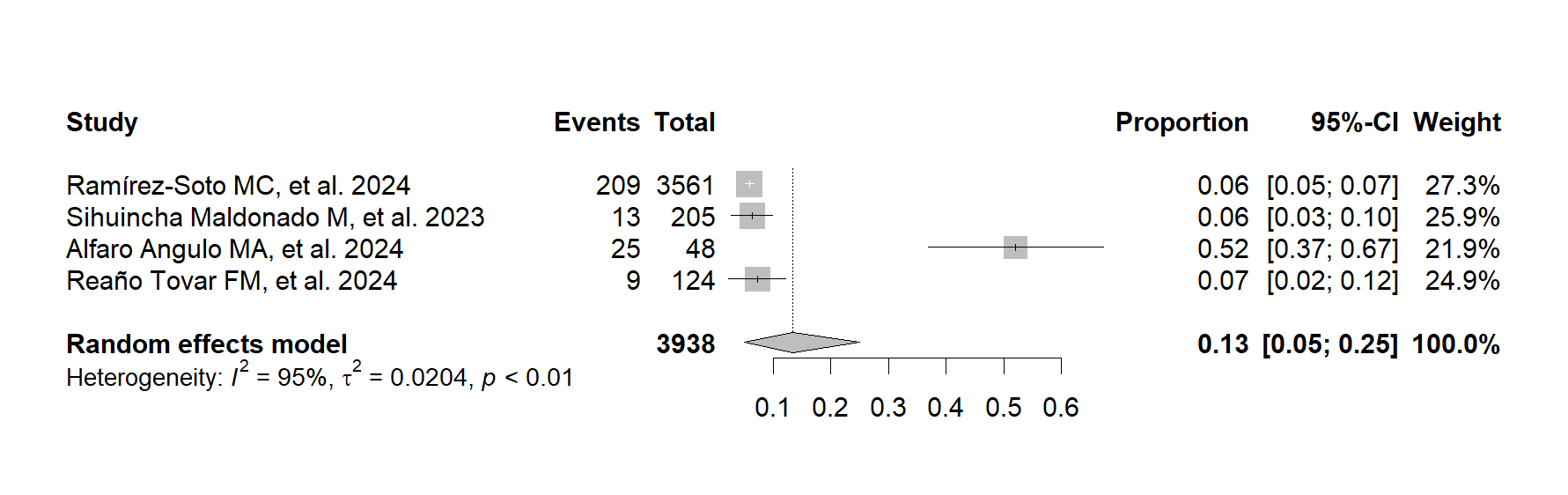

Supplement: S15 Fig — (TIF) [file pone.0327097.s021.tif]

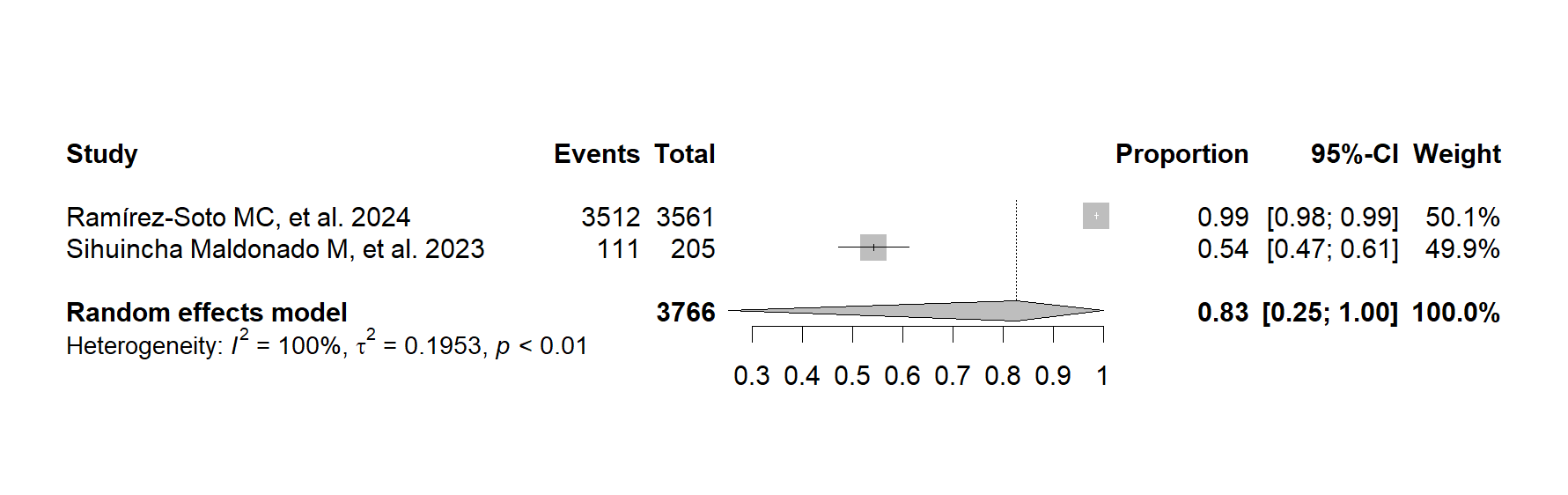

Supplement: S16 Fig — (TIF) [file pone.0327097.s022.tif]

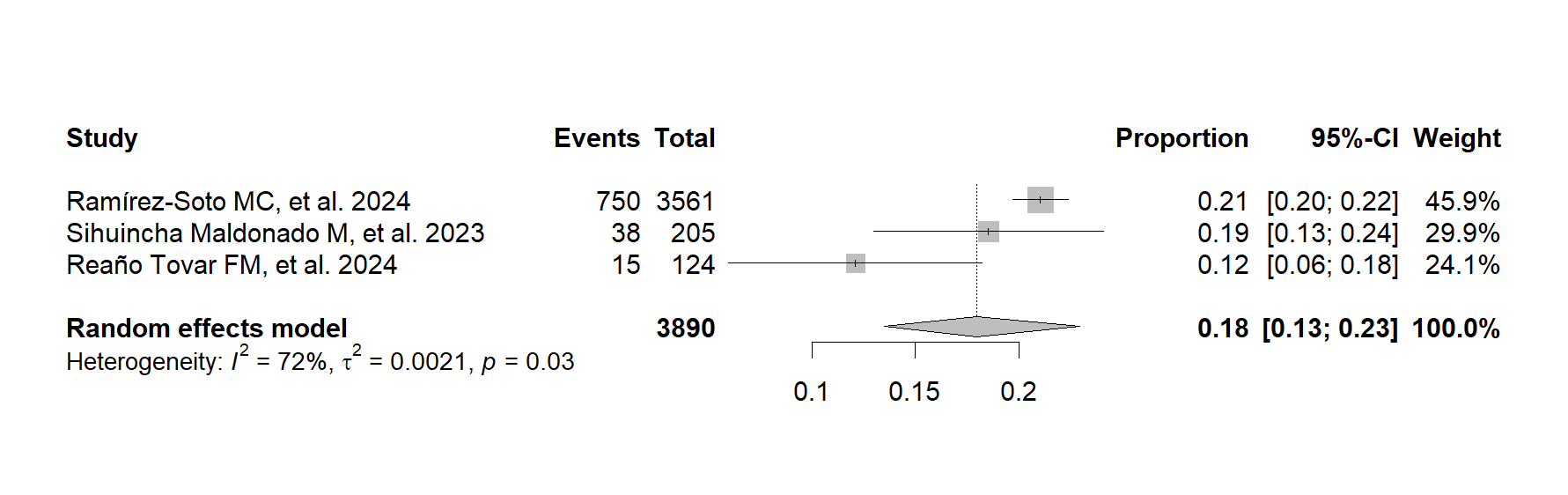

Supplement: S17 Fig — (TIF) [file pone.0327097.s023.tif]

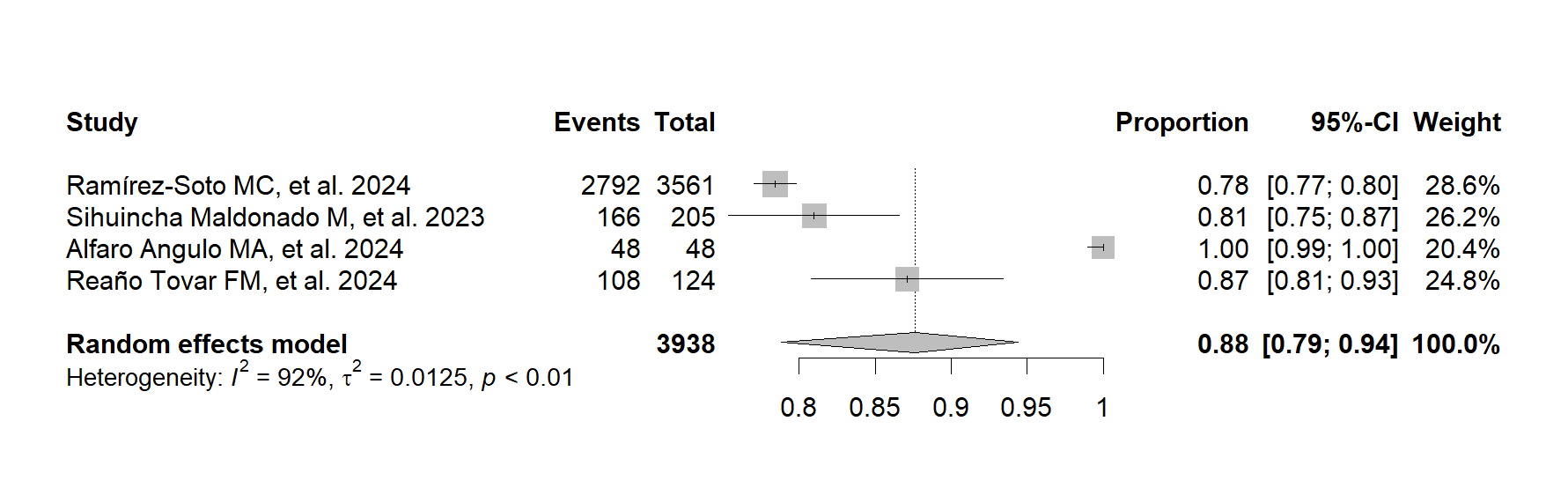

Supplement: S18 Fig — (TIF) [file pone.0327097.s024.tif]

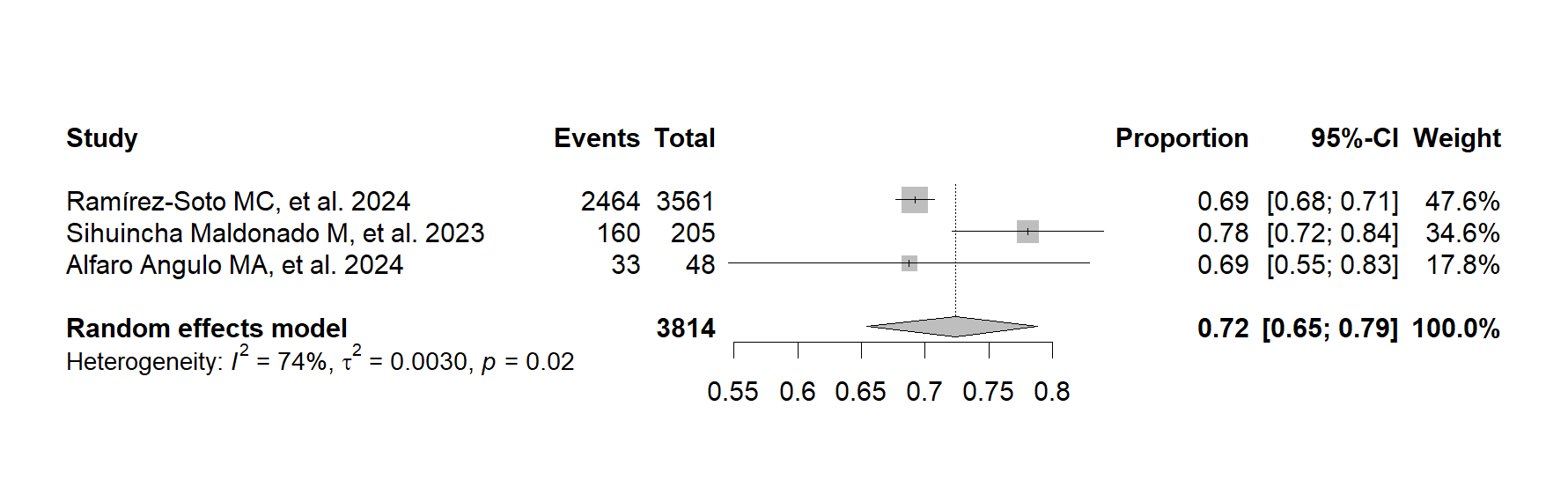

Supplement: S19 Fig — (TIF) [file pone.0327097.s025.tif]

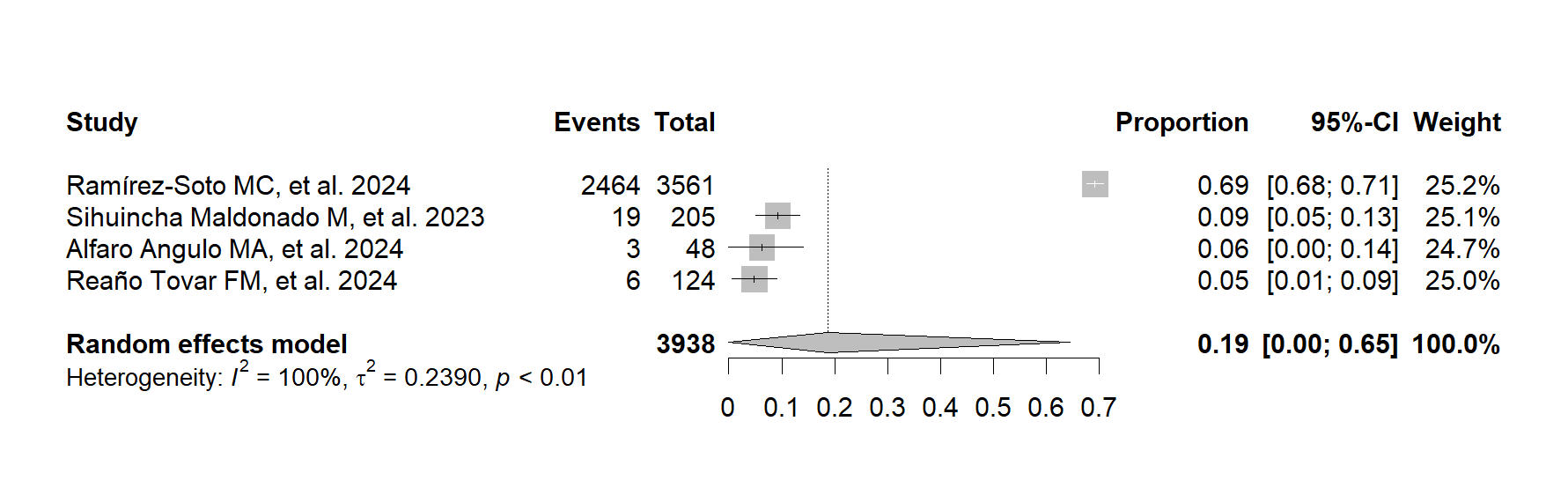

Supplement: S20 Fig — (TIF) [file pone.0327097.s026.tif]
