# Supplementary material for: Epidemiological and clinical characteristics of Peruvian patients with mpox: A systematic review and meta-analysis
Source: PLoS One. 2025 Jun 25;20(6):e0327097. doi: 10.1371/journal.pone.0327097 (PMC12194101; doi:10.1371/journal.pone.0327097)
Supplement: Attachment — Submitted filename: Response to Reviewers.pdf [file pone.0327097.s027.pdf]

Dear **Editor**,

Thank you very much for reviewing our article, "Epidemiological and clinical characteristics of Peruvian patients with mpox: a systematic review and meta-analysis". Your suggestions and comments will be addressed below. Thank you for your valuable time and excellent review.

Editor's comments

1. 1. When submitting your revision, we need you to address these additional requirements. Please ensure that your manuscript meets PLOS ONE's style requirements, including those for file naming. The PLOS ONE style templates can be found at https://journals.plos.org/plosone/s/file?id=wjVg/PLOSOne_formatting_sample_main_body.pdf and https://journals.plos.org/plosone/s/file?id=ba62/PLOSOne_formatting_sample_title_authors_affiliations.pdf

Our response: Thank you for your feedback. We have reviewed and adjusted our manuscript in accordance with the guidelines provided by PLOS ONE. Additionally, we have ensured that all files meet the required formatting standards, including the proper file naming.

Attached is the revised version of the manuscript, now aligned with the specified guidelines, including the correct format for the title, authors, affiliations, and the main body of the article.

2. 2. We notice that your supplementary figures are uploaded with the file type 'Figure'. Please amend the file type to 'Supporting Information'. Please ensure that each Supporting Information file has a legend listed in the manuscript after the references list.

Our response: Thank you for your comments. We have updated the file type to "Supplementary Information," as requested.

Additionally, we have ensured that each supplementary file is referenced in the manuscript. The captions for each figure are not specified separately, as they are included in full within the main text of the article.

3. 3. When completing the data availability statement of the submission form, you indicated that you will make your data available on acceptance. We strongly recommend all authors decide on a data sharing plan before acceptance, as the process can be lengthy and hold up publication timelines. Please note that, though access restrictions are acceptable now, your entire data will need to be made freely accessible if your manuscript is accepted for publication. This policy applies to all data except where public deposition would breach compliance with the protocol approved by your research ethics board. If you are unable to adhere to our open data policy, please kindly revise your statement to explain your reasoning and we will seek the editor's input on an exemption. Please be assured that, once you have provided your new statement, the assessment of your exemption will not hold up the peer review process.

Our response: We have modified the data availability statement. All our data are included in the article and its supplementary materials.

4. 4. As required by our policy on Data Availability, please ensure your manuscript or supplementary information includes the following:

Our response: The availability of the data was verified, and it is detailed in the article and supplementary material.

5. A numbered table of all studies identified in the literature search, including those that were excluded from the analyses.

Our response: The numbered table of all the studies identified in the bibliographic search has been presented. Please verify S4 Table.

6. For every excluded study, the table should list the reason(s) for exclusion.
Our response: The excluded studies have been presented along with the reasons for exclusion. Please verify S4 Table.

7. If any of the included studies are unpublished, include a link (URL) to the primary source or detailed information about how the content can be accessed.
Our response: The included studies were verified, and all are published.

S4 Table

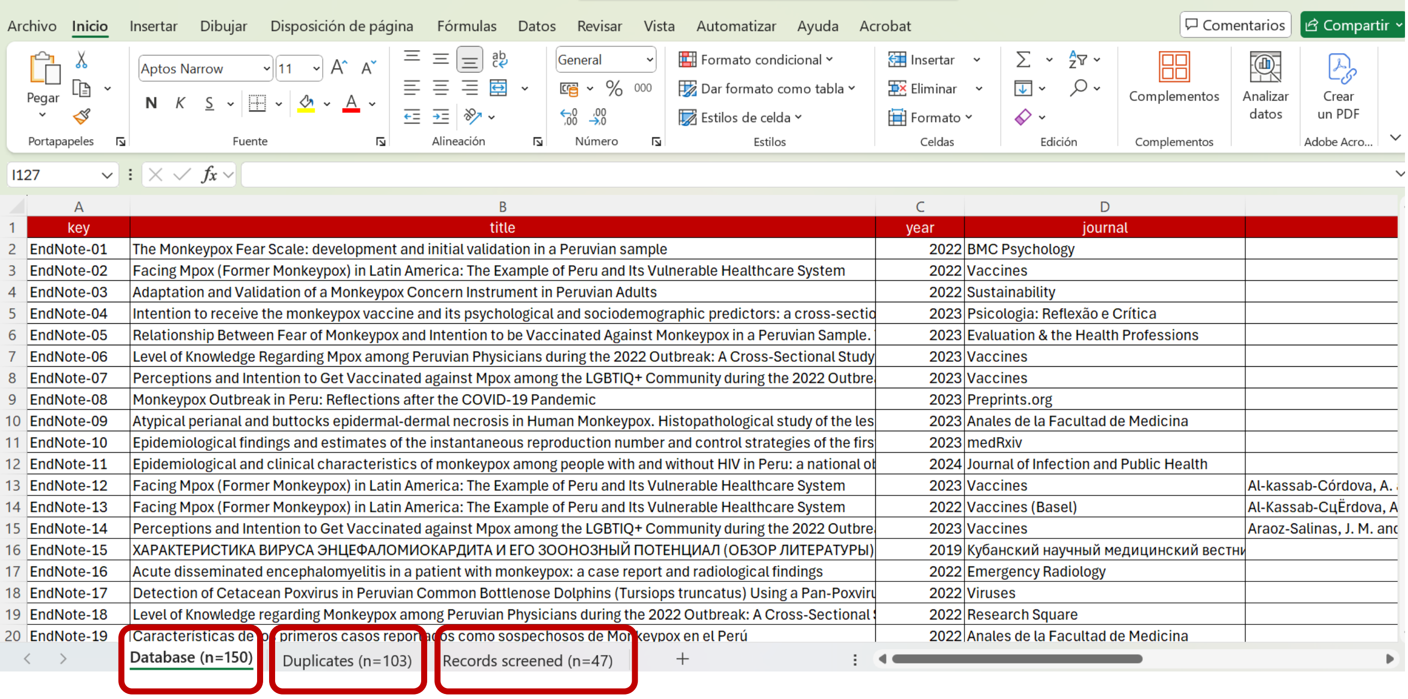

	key	title	year	journal	
2	EndNote-01	The Monkeypox Fear Scale: development and initial validation in a Peruvian sample	2022	BMC Psychology	
3	EndNote-02	Facing Mpox (Former Monkeypox) in Latin America: The Example of Peru and Its Vulnerable Healthcare System	2022	Vaccines	
4	EndNote-03	Adaptation and Validation of a Monkeypox Concern Instrument in Peruvian Adults	2022	Sustainability	
5	EndNote-04	Intention to receive the monkeypox vaccine and its psychological and sociodemographic predictors: a cross-sectio	2023	Psicologia: Reflexão e Crítica	
6	EndNote-05	Relationship Between Fear of Monkeypox and Intention to be Vaccinated Against Monkeypox in a Peruvian Sample.	2023	Evaluation & the Health Professions	
7	EndNote-06	Level of Knowledge Regarding Mpox among Peruvian Physicians during the 2022 Outbreak: A Cross-Sectional Study	2023	Vaccines	
8	EndNote-07	Perceptions and Intention to Get Vaccinated against Mpox among the LGBTIQ+ Community during the 2022 Outbre	2023	Vaccines	
9	EndNote-08	Monkeypox Outbreak in Peru: Reflections after the COVID-19 Pandemic	2023	Preprints.org	
10	EndNote-09	Atypical perianal and buttocks epidermal-dermal necrosis in Human Monkeypox. Histopathological study of the les	2023	Anales de la Facultad de Medicina	
11	EndNote-10	Epidemiological findings and estimates of the instantaneous reproduction number and control strategies of the firs	2023	medRxiv	
12	EndNote-11	Epidemiological and clinical characteristics of monkeypox among people with and without HIV in Peru: a national ob	2024	Journal of Infection and Public Health	
13	EndNote-12	Facing Mpox (Former Monkeypox) in Latin America: The Example of Peru and Its Vulnerable Healthcare System	2023	Vaccines	Al-kassab-Córdova, A.
14	EndNote-13	Facing Mpox (Former Monkeypox) in Latin America: The Example of Peru and Its Vulnerable Healthcare System	2022	Vaccines (Basel)	Al-Kassab-CцЁrdova, A
15	EndNote-14	Perceptions and Intention to Get Vaccinated against Mpox among the LGBTIQ+ Community during the 2022 Outbre	2023	Vaccines	Araoz-Salinas, J. M. and
16	EndNote-15	ХАРАКТЕРИСТИКА ВИРУСА ЭНЦЕФАЛОМИОКАРДИТА И ЕГО ЗООНОЗНЫЙ ПОТЕНЦИАЛ (ОБЗОР ЛИТЕРАТУРЫ)	2019	Кубанский научный медицинский вестни	
17	EndNote-16	Acute disseminated encephalomyelitis in a patient with monkeypox: a case report and radiological findings	2022	Emergency Radiology	
18	EndNote-17	Detection of Cetacean Poxvirus in Peruvian Common Bottlenose Dolphins (Tursiops truncatus) Using a Pan-Poxviru	2022	Viruses	
19	EndNote-18	Level of Knowledge regarding Monkeypox among Peruvian Physicians during the 2022 Outbreak: A Cross-Sectional S	2022	Research Square	
20	EndNote-19	Características de [illegible] primeros casos repor[illegible]os como sospechosos de Mo[illegible]keypox en el Perú	2022	Anales de la Facultad de Medicina	

8. A table of all data extracted from the primary research sources for the systematic review and/or meta-analysis. The table must include the following information for each study:
Our response: Your recommendation is included in the article and supplementary material. Please review Tables 2, 3, 4, and S5 Table.

9. Name of data extractors and date of data extraction
Our response: The initials of the data extractors and the date of data extraction were added.

10. Confirmation that the study was eligible to be included in the review.
Our response: Eligibility criteria were established to confirm that the study met the requirements to be included in the review.

11. All data extracted from each study for the reported systematic review and/or meta-analysis that would be needed to replicate your analyses.
Our response: All extracted data, as well as the included and excluded articles, are reported in detail in the study and supplementary material. It is recommended to review

the documents and tables provided. Our study can be replicated, as it presents the R codes used in detail, along with tables containing the data entered into the analysis.

12. If data or supporting information were obtained from another source (e.g. correspondence with the author of the original research article), please provide the source of data and dates on which the data/information were obtained by your research group.
    Our response: All supporting information and data are detailed in the supplementary material and do not require permission, as they were created by the authors based on published articles.

13. If applicable for your analysis, a table showing the completed risk of bias and quality/certainty assessments for each study or outcome. Please ensure this is provided for each domain or parameter assessed. For example, if you used the Cochrane risk-of-bias tool for randomized trials, provide answers to each of the signalling questions for each study. If you used GRADE to assess certainty of evidence, provide judgements about each of the quality of evidence factor. This should be provided for each outcome.
    Our response: Our study and its analysis include a table that presents the complete risk of bias and quality/uncertainty assessments for each study or outcome using the JBI tool. Additionally, publication bias was assessed using a funnel plot and the Egger test. Review supplementary material.

14. An explanation of how missing data were handled.
    Our response: All the data extracted from the articles are presented in detail in the main article and supplementary material.

15. This information can be included in the main text, supplementary information, or relevant data repository. Please note that providing these underlying data is a requirement for publication in this journal, and if these data are not provided your manuscript might be rejected.
    Our response: We agree with your comment. All the data have been provided in the article and supplementary materials.

16. Additional Editor Comments: I commend the authors for their work. It was a good approach to stratify the data by the publication year. However, there are some concerns raised by the reviewers. These should be properly addressed before further consideration.
    Our response: Thank you for your valuable comments. We have addressed all the reviewers' observations and suggestions in detail, ensuring that the necessary improvements have been incorporated into the document.

17. In addition to these comments, please address the following: 1- Why are all the figures provided as supplementary S1-S2-S3, etc.?
    Our response: The figures in the supplementary material (S1, S2, S3, etc.) provided serve as evidence for the synthesis in Table 5. These figures support the prevalence of the epidemiological and clinical characteristics of Peruvian patients with mpox.
    The author guidelines specify a set number of figures, which is why they were included in the supplementary material.

18. 2- There is a considerable amount of heterogeneity in most analyses. Have you considered doing sensitivity analyses? Galbraith plots? checking for outliers?

Our response: We appreciate your comment regarding the considerable heterogeneity observed in the analyses. We agree with your observation and have addressed this heterogeneity in the limitations of the study, providing an explanation for it. Although we considered conducting a sensitivity analysis, it was not feasible due to the variability in the available data.

Nevertheless, we established strict criteria for our systematic review, following PRISMA guidelines. Additionally, we developed a detailed protocol, devised a rigorous search strategy, and employed methods to assess the quality of evidence and publication bias.

Dear Editor, all reviewer comments have been addressed. The English grammar has been improved, and the article has been formatted according to the specific guidelines of Plos One. Additionally, the epidemiological data has been updated in line with the latest information from the World Health Organization (WHO).

Reviewer #1:

Overall assessment;
This manuscript has attempted to provide a comprehensive analysis of the epidemiological and clinical characteristics of mpox in Peruvian patients. While the study contributes valuable data to the growing body of literature on mpox in Latin America, there are several critical issues regarding methodological rigor, clarity of discussion, and the interpretation of findings. Below is a detailed evaluation of the manuscript's strengths and weaknesses.
Our response: We greatly appreciate your review of our article, dear doctor. We have addressed all the observations and accepted the recommendations provided.

1. **Reviewer says:** 1. Methodological limitation
   • The retrospective nature of most included studies is acknowledged but not critically analyzed in areas such as recall bias, missing data, and confounding variables that could have influenced the reported epidemiological trends.
   Our response: We deeply appreciate the reviewer's observation and fully agree with it. We acknowledge that most of the included studies have a retrospective design, which introduces several factors that could have influenced the observed results and epidemiological trends. In fact, we assessed the quality of the included studies, which allowed us to identify potential limitations inherent to these designs.

   In the study limitations section, we have highlighted these critical aspects, particularly recall bias, missing data, and confounding variables. Recall bias may have affected the accuracy of the data, as participants or retrospective records could have had difficulties accurately remembering past events. Additionally, missing data (e.g., loss to follow-up or incomplete information) may have introduced further biases that impacted the validity of the results. Finally, confounding variables, such as uncontrolled or inadequately identified factors in the studies, could have influenced the observed trends and distorted the conclusions.

2. **Reviewer says:** 2. Overgeneralization and lack of nuanced interpretation
   • The authors heavily emphasize that the predominance of mpox cases in men, particularly MSM, is consistent with global trends. However, the discussion lacks a critical analysis of potential underreporting or diagnostic biases among women and other

populations. Are women less affected, or is their presentation underrecognized and underreported?

Our response: Thank you for your valuable observation. In response, we have added a paragraph in the Discussion section that addresses the potential underreporting and diagnostic biases affecting women and other populations. The revised text acknowledges that while current data indicate a predominance of mpox cases among men, particularly MSM, this pattern may not reflect the actual burden in other groups. We now emphasize the importance of inclusive surveillance strategies and a more nuanced interpretation of the data to better capture the epidemiology of mpox across diverse populations.

3. **Reviewer says:** •The claim that transgender women are at higher risk than cisgender women lack adequate contextualization. The authors cite studies suggesting differential risks but do not explore how societal, behavioral, and healthcare access factors contribute to these disparities.

   Our response: Thank you for your comments. We have reviewed and modified the manuscript to more thoroughly address the societal, behavioral, and healthcare access factors contributing to the disparities in infection risks between transgender women and cisgender women. Following your recommendations, we expanded the discussion based on the cited articles, explaining how social stigma, discrimination, the higher prevalence of HIV in transgender women, and barriers to healthcare access contribute to their increased vulnerability to mpox infection. We believe these changes provide better contextualization of the elevated risk faced by transgender women and offer a more comprehensive analysis of the factors influencing these disparities.

4. **Reviewer says:** •The assertion that mpox severity is strongly correlated with HIV status is valid but lacks differentiation between well-controlled HIV infection and advanced HIV/AIDS. Instead of using ART access as a measure of how controlled HIV infection is (which may be flawed if viral load is higher), the discussion can be more robust by explicitly distinguishing between HIV+ individuals with undetectable viral loads (or a categorical spectrum thereof) and those with significant immunosuppression (low CD4/high viral load).

   Our response: Thank you for your valuable comments. In response to your observation, we have reorganized and expanded the discussion following your recommendations. An explicit distinction has been made between individuals with well-controlled HIV, characterized by undetectable viral loads and adequate CD4 counts, and those with advanced HIV or AIDS, who exhibit significant immunosuppression, marked by low CD4 counts and high viral loads. This revision makes the discussion on the correlation between mpox severity and HIV status more accurate and robust, addressing the impact of immunosuppression on the clinical course of the disease.

5. **Reviewer says:** 3. Inconsistencies in clinical data reporting:

   •The discussion on hospitalization rates compares the Peruvian cohort (5%) with a Brazilian study (10.5%) without acknowledging potential confounders such as differences in healthcare infrastructure, diagnostic criteria, and thresholds for hospitalization.

   Our response: Thank you for your valuable feedback. In response to your observation, we have revised the discussion to address potential confounders that may explain the differences in hospitalization rates between the Peruvian and Brazilian cohorts. We acknowledge that factors such as differences in healthcare infrastructure, diagnostic criteria, and thresholds for hospitalization could influence these rates.

6. **Reviewer says:** 4. Citations issues:
•The manuscript cites multiple systematic reviews and meta-analyses (e.g., Ortiz-Saavedra et al., Benites-Zapata et al…etc), but the authors do not critically engage with these studies' limitations, such as sample heterogeneity or publication bias.
Our response: Thank you for your valuable comments. We have added and highlighted the recommended changes, acknowledging that it is crucial to consider the limitations of the cited studies, such as sample heterogeneity due to variations in populations, regions, and clinical settings, which can affect the generalizability of the results. Additionally, we addressed the potential publication bias, which could overrepresent studies with positive results, influencing the overall conclusions. These considerations have been incorporated into the discussion to provide a more nuanced and accurate view of the findings.

7. **Reviewer says:** 1. Lack of clarity in writing and organization:
•Some sections contain redundant information. For example, the discussion of HIV comorbidity appears in multiple parts of the text, leading to somewhat unnecessary overemphasized repetition.
Our response: Thank you for your comments. In response to your observation, we have reviewed and revised the manuscript to improve clarity and organization, eliminating redundancies in the discussion of HIV comorbidity. The information has been restructured to avoid unnecessary repetition, consolidating relevant sections and presenting the key points in a more coherent manner. We believe these changes have enhanced the flow of the text and the clarity of the findings.

8. **Reviewer says:** •The manuscript would benefit from clearer subheadings within the discussion section to separate epidemiological trends, clinical presentation, and disease severity.
Our response: Thank you for your valuable comments. We have taken your recommendation into account and reorganized the discussion section to include clear subheadings that better separate the topics addressed. Specifically, we have added subheadings dedicated to epidemiological trends, clinical presentation, and disease severity. We believe this structure will enhance the clarity of the manuscript and facilitate the understanding of the topics discussed in this section.

9. **Reviewer says:** 2. Limited discussion on public health implications:
•While the study discusses clinical manifestations, it does not sufficiently explore public health interventions. How should these findings inform mpox prevention strategies in Peru? What are the implications for vaccination, contact tracing, and community engagement?
Our response: Thank you for your valuable comments. In response to your observation regarding the limited discussion of public health implications, we have added a section that addresses key prevention strategies and interventions in the context of mpox, particularly in populations with high HIV prevalence. This new discussion emphasizes the importance of vaccination, contact tracing, and community engagement as essential components for controlling the spread of the disease in Peru. We believe these considerations will enhance the applicability and relevance of the findings in the public health context.

10. **Reviewer says:** •The manuscript briefly mentions that "intervention studies focused on prevention and treatment in HIV co-infected patients could also offer key strategies to mitigate the effects of future outbreaks." This is a crucial point that deserves further expansion.
    Our response: Thank you very much for your observation and suggestion. We have taken your comment into account regarding the importance of expanding the section that mentions intervention studies focused on prevention and treatment in HIV co-infected patients. In response, we have expanded this section, incorporating additional details on potential key strategies to mitigate the effects of future outbreaks, as well as the relevance of these studies for the development of therapeutic and preventive approaches tailored to the specific needs of this population.

11. **Reviewer says:** •The conclusions should be refined to offer more actionable public health recommendations. The conclusion that these findings support prevention strategies is overly broad and lacks specificity. What exact strategies should be prioritized based on this data?
    Our response: Thank you for your valuable feedback. In response to your observation, we have refined the conclusions to offer more actionable public health recommendations. We have expanded the section by specifying targeted prevention strategies, including prioritizing efforts for high-risk populations, the integration of routine HIV testing and mpox screening, educational campaigns, and the strengthening of healthcare infrastructure. These strategies are now detailed in the revised conclusion to better align with the data presented.

12. **Reviewer says:** Future directions
    •The call for longitudinal studies is appropriate, but the authors should specify which knowledge gaps remain unaddressed.
    Our response: It was organized as suggested, and the existing knowledge gaps were reflected.

13. **Reviewer says:** Other comments.
    1.Confidence intervals are still wide for some estimates (thanks for sensitivity analysis), yet their implications are not discussed. For example, the estimate for lymphadenopathy is 83% (95% CI: 25-100%), making these pooled results highly questionable.
    Our response: Thank you for your valuable feedback. We appreciate your observation regarding the wide confidence intervals for some estimates, particularly for lymphadenopathy, where the 95% confidence interval ranges from 25% to 100%. We recognize that such wide intervals may introduce uncertainty into the interpretation of the pooled results.

    In response, we have added a discussion of the implications of these wide confidence intervals in the manuscript. Specifically, we highlight the need for caution when interpreting estimates with high variability and acknowledge that further studies with larger sample sizes and more precise measurements are necessary to reduce uncertainty and provide more reliable estimates. We also discuss the potential impact of this variability on the overall findings and the importance of considering these factors when drawing conclusions.

14. **Reviewer says:** 2.S-table 3 talks about the quality of the dengue studies included in the review. This seems unusual as there is no relevance to dengue for this paper. An explanation could help the reader to understand.
Our response: A thousand apologies for this. The wording of the title has been corrected.

15. **Reviewer says:** 3.The choice of databases includes some that are not standard for systematic reviews (e.g., Google Scholar, which lacks robust indexing and reproducibility). The inclusion of these databases should be justified.
Our response: Thank you for your comment. We appreciate your concern regarding the inclusion of databases like Google Scholar, which may not be considered standard for systematic reviews. We acknowledge the limitations of using this database, particularly in terms of indexing and reproducibility. We have reviewed the manuscript, which indicates that the search strategy was conducted in databases and search tools due to their usefulness in capturing a broader range of studies, particularly publications that may not be indexed in more traditional databases. However, we also recognize the importance of reproducibility and have ensured that our methodology includes more standardized databases to maintain the rigor of the review. We believe this balanced approach enhances the comprehensiveness of the review while addressing your valid concerns. Thank you again for pointing this out.

16. **Reviewer says:** My recommendation is that this manuscript provides valuable epidemiological insights into mpox in Peru. However, substantial revisions are necessary to enhance methodological transparency, refine the discussion of key findings, and strengthen the manuscript's public health implications. Addressing these concerns will significantly improve its scientific rigor and impact. Specifically, by address the raised issues above including refining the discussion, ensuring more rigorous citation practices, improving clarity and structure, and expanding the public health relevance will enhance the manuscript's contribution to the infectious disease research community.
Our response: Thank you for your thoughtful and constructive feedback. We greatly appreciate your recognition of the valuable epidemiological insights provided in the manuscript. We have carefully addressed all of your suggestions in detail. Specifically, we have enhanced the methodological transparency, refined the discussion of key findings, and ensured more rigorous citation practices throughout the manuscript. We have also improved the clarity and structure of the manuscript to enhance its readability. Additionally, we have expanded the public health implications to further strengthen the relevance of the study. We are confident that these revisions significantly improve the scientific rigor and impact of the manuscript. Thank you again for your invaluable input.

Reviewer #2:

The article shows important descriptive data that can be useful to understand the characteristics of mpox in the context of Peru and Latin American. However, I consider that some adjustments are necessary prior to publication.
Our response: Thank you for your valuable feedback. We appreciate your recognition of the importance of the descriptive data presented in our article, and we are glad it can be useful for understanding the characteristics of mpox in the context of Peru and Latin America. We acknowledge your suggestion for adjustments and will carefully review the manuscript to address the points raised. We will make the necessary revisions to improve

clarity and ensure the article meets the required standards for publication. Thank you again for your constructive comments.

1. **Reviewer says:** 1) Abstract
"The main objective of this study is to determine the prevalence of epidemiological and clinical characteristics of Peruvian patients diagnosed with mpox, providing a detailed view of the situation of this affected population."
I suggest changing this phrase to "The main objective of this study is to determine the epidemiological and clinical characteristics of Peruvian patients diagnosed with mpox, providing a detailed view of the situation of this affected population."
Our response: Thank you for your suggestion. The indicated correction has been made to the abstract to more accurately reflect the study's objective.

2. **Reviewer says:** 2) Introduction
The data of Table 1 don't add much to the Introduction where they are currently placed. The characteristics described are not mentioned in the Introduction and neither in the Discussion. It would be interesting to compare them with the results of the author's review in the Discussion.
Our response: Thank you very much for your valuable comments. In response to your suggestion, we have added a detailed description of Table 5 in the Discussion, where we compare our findings with the results of other relevant studies. We hope this addition provides clarity and context to the presented results.

3. **Reviewer says:** 3) Methods
- Explain the reason for excluding randomized clinical trials of the review.
Our response: Thank you for your comment. Randomized clinical trials were excluded from our review because their experimental design does not align with the focus of our systematic review, which is centered on observational studies. Additionally, it is important to note that in Peru, where the review was conducted, the implementation of clinical trials is limited by factors such as high costs and long durations, making them less common in local research.

However, in the Discussion, we recommend conducting higher-quality studies, such as clinical trials, and emphasize the importance of more thorough follow-up on the characteristics of mpox. We believe that these studies would be essential for improving the understanding of the disease and its long-term implications.

4. **Reviewer says:** 4) Results
- The titles of Tables 2 and 3 should inform that they are presenting data from the observational studies. This is important because the data of the case series and case reports are presented separately in Table 4. The titles of the tables should allow understanding independently, without the necessity of referring to the main text.
Our response: Thank you very much for your comment. We have reviewed the titles of the tables included in the article and updated each one to clearly and concisely reflect the clinical or epidemiological characteristics presented in the meta-analysis or systematic review.
Observational studies are presented in a separate table because they provide global data on the participants included in the review. On the other hand, case reports and case series are organized in a different table, as they provide specific information about each patient.

This organization was determined to make the information more accessible to the audience.
The titles of the tables have been adjusted to align with the objectives of the study, and as it is a systematic review, it is clearly stated that these are summaries of the included studies.
Once again, thank you for your excellent comment. If you have any suggestions regarding the titles of the tables, the authors would be happy to review and adjust them as needed.

5. **Reviewer says:** "On the other hand, the fact that 91% of coinfected patients were receiving antirretroviral therapy suggests that, although access to treatment is adequate, these individuals remain susceptible to mpox infection, raising questions about the impact of relative immunosuppression on the clinical course of the disease."
I suggest rephrasing this paragraph. The presence of HIV coinfection does not change the susceptibility to mpox infection, since several immunocompetent individuals were also infected. If severe immunosuppression is present, it can alter the severity of mpox, but the study does not address outcomes. Hospitalization is discussed in later paragraphs, what I consider important to maintain. Possible reasons for the finding of the high prevalence of HIV coinfection among the mpox cases should be discussed.
Our response: The paragraph was rephrased, and published articles on HIV coinfection and its association with susceptibility to mpox infection were reviewed. Additionally, the discussion on hospitalization was expanded, and the possible reasons for the high prevalence of HIV coinfection among mpox cases were explored.

6. **Reviewer says:** - Data from the observational studies were analyzed separately from the ones of the case series and reports. I understand from reading the manuscript that individual data were not available for the four observational studies included in the review. If this assumption is correct, this should be stated in the text or, if it's not, the reason why pooled individual data analysis was not performed in variables such as sex or HIV status.
Our response: Thank you very much for your comment. In the methodology, the data were organized based on the meta-analysis as well as the case reports and series. The data from the observational studies (global data used for the meta-analysis) were analyzed separately from the data from the case series and reports (specific patient data presented descriptively).

This distinction was outlined in the strengths and limitations section of the study, with the aim of emphasizing the need for further research, not only observational but also experimental, including long-term follow-up.

If you have any comments or recommendations, we are ready to respond.

Sincerely,

Mario J. Valladares-Garrido

Universidad Continental, Lima 15046, Peru; mvalladares@continental.edu.pe
